# Understanding mental health stigma and discrimination in Ethiopia: A qualitative study

**DOI:** 10.1017/gmh.2024.55

**Published:** 2024-04-30

**Authors:** Eshetu Girma, Bethel Ayele, Petra C. Gronholm, Syed Shabab Wahid, Ariam Hailemariam, Graham Thornicroft, Charlotte Hanlon, Brandon Kohrt

**Affiliations:** 1 Department of Preventive Medicine, School of Public Health, Addis Ababa University, Addis Ababa, Ethiopia; 2 Centre for Global Mental Health and Centre for Implementation Science, Institute of Psychology, Psychiatry & Neuroscience, King’s College London, London, UK; 3 Department of Global Health, Georgetown University, Washington DC, USA; 4 Centre for Global Mental Health, Health Service and Population Research Department and WHO Collaborating Centre for Mental Health Research and Training, Institute of Psychiatry, Psychology and Neuroscience, King’s College London, London, UK; 5 Department of Psychiatry, School of Medicine, College of Health Sciences, Addis Ababa University, Addis Ababa, Ethiopia; 6 Center for Global Mental Health Equity, The George Washington University, Washington DC, USA

**Keywords:** Mental conditions, Stigmatization, Discrimination, low and middle income country, Ethiopia

## Abstract

**Background:**

Stigma is significantly impacted by cultural and contextual value systems. People with mental health conditions frequently have to deal with the condition itself and the associated stigma and discrimination. Contextual understanding is essential to design measures and interventions.

**Objective:**

This study aimed to explore the experiences and perceptions of people with mental health conditions, their families and key stakeholders.

**Method:**

A qualitative method used to understand mental health-related stigma and its local contexts. Sixteen participants, including service users, caregivers, service providers and health service administrators, were interviewed.

**Result:**

People with mental health conditions and their caregivers experienced various forms of stigmatization which is linked to attributions about the causality of the illness, overt manifestations of mental health condition leading to easy identification and functional impairments that adversely affect participation. Social contact, lived experiences sharing and training of service providers are relevant intervention strategy to address stigma.

**Implication:**

Stigma and exclusion are prominent in the experiences of people with mental health conditions and their caregivers in this rural Ethiopian setting. Measurement of stigma and the development of interventions should consider how stigma is socially constructed. Anti-stigma interventions need to be implemented alongside expanded local access to mental healthcare.

## Impact statement

People with mental health conditions (MHCs) face major challenges due to negative stereotypes and prejudice generated by misconceptions about MHCs. While research has gone a long way toward understanding the multiple adverse impacts of MHCs in low- and middle-income countries (LMICs), it is just starting to explain the social construct of mental health-related stigma. Contextualization of stigma measures and development of anti-stigma programs in LMICs are still limited. This study offers a valuable perspective on the experience of stigma and the mechanisms underlying the stigmatization of individuals with mental illnesses and key stakeholders. This enables a thorough investigation of the local context, focusing on the viewpoints and understandings of persons who reside and work in the area. That also looked at the stigma associated with mental health issues more broadly than from the perspective of a particular illness or diagnosis. The present study can be effective in targeting the consequences of stigma and understanding the interpretation of this stigma can aid in the development of context-specific anti-stigma interventions in Ethiopia and provides input into the process of assessing the implications and concept of stigma at a cross-national level. These interventions endorse the use of social contact approaches to reduce stigma, which are acceptable as long as they are combined with measures that foster a supportive environment, such as increased availability of mental health services.

## Introduction

The stigma associated with mental health conditions (MHCs) remains a significant social issue, with no nation or culture placing the same value on people with MHCs as those without (Rössler, 2016). It has been reported by those who have been impacted that stigma can be worse than the MHC itself (Shrivastava et al., 2012; Thornicroft et al., 2022). Stigma may have a variety of adverse impacts on social inclusion, well-being, employment opportunities, poverty and relationships (Hanlon, 2017), as well as impeding healthcare seeking behaviors and contributing to the nonavailability of quality mental healthcare (Pescosolido and Martin, 2015; Subu et al., 2021). The idea of stigma can be divided into two categories: action-oriented: public stigma (The general public’s endorsement of prejudice and discrimination), structural stigma (discrimination via laws, regulations and constitutional norms), courtesy stigma (discrimination, and preconceptions that are gained through relationships with stigmatized individuals or groups), provider-based stigma (prejudice and discrimination by professional associations assigned to support marginalized communities) and self-stigma (when the stigmatized group accept the public stigma as true and internalized to their own lives) which generally specify who or what gives or gets the stigma and experiential: perceived (a viewpoint that “the majority of people” believe to have), endorsed (indicating support for discrimination, prejudice and stereotypes), anticipated (awaiting experiences with prejudice and discrimination), received (direct encounters with devaluation or rejection) and enacted stigma (displaying prejudiced actions/behavior) that explain how stigma arises (Gronholm et al., 2017).

There has been increased research interest in developing and evaluating interventions that seek to address stigma and discrimination against people with MHCs (Clay et al., 2020). Systematic reviews of the efficacy of such interventions indicate that education-based interventions (addressing myths and misconceptions) and social contact-based interventions involving direct or indirect interactions with people who have the stigmatized condition) have small to moderate effect sizes on stigma reduction in the short- to medium-term (Waqas et al., 2020; Makhmud et al., 2022). In a recent review of studies from low- and middle-income countries (LMICs), several approaches to reducing stigma related to MHC had been evaluated, but social contact was barely used even though the evidence base is strongest for this approach. Existing studies from LMICs are limited by methodologically weak research designs and minimal use of local expertise. Furthermore, the authors found that studies were restricted to a limited number of LMICs (Clay et al., 2020).

The effectiveness of interventions designed to lessen stigma and discrimination in the local community has not received much attention from research studies published in LMICs (Kapungwe et al., 2010; Thornicroft et al., 2022). Despite the fact that programs working with marginalized or stigmatized groups frequently include community awareness-raising, there is a shortage of evidence regarding whether awareness-raising strategies alone are effective in reducing stigma in the community, especially with regard to changes beyond knowledge, covering the crucial areas of attitudes and behavior (Thornicroft et al., 2007). Other strategies, such as social contact interventions, have been shown to be one of the most effective ways to promote behavior change, as evidenced by a decrease in the use of discriminatory practices by community members (Thornicroft et al., 2016; Shahwan et al., 2022).

In Ethiopia, people still viewed mental health problems as curses from God (Zeleke et al., 2019). Ethiopians have a distinct cultural and traditional understanding of mental illness that primarily links it to spiritual origins, disruptions in the divine-human relationship, and the “curse” or punishment of God on wrong dowers (Jacobsson and Merdasa, 1991; Zeleke et al., 2019). Mental illnesses were associated with psychocultural inappropriateness or engaging in taboo (Monteiro and Balogun, 2014). The idea of what causes mental illness and how society characterizes those people with MHC creates stigma. This is due to the fear that individuals with mental illness may harm others (Yeshanew et al., 2020).

In order to successfully reduce stigmatization and improve the experience of service users, anti-stigma interventions need to be developed, modified and implemented in a specific local context. Analyzing the various kinds of stigma and identifying the specific groups affected is essential for developing effective strategies for lowering stigma and prejudice (Gronholm et al., 2017). A synthesis of evidence on mental health stigma and discrimination in Ethiopia highlighted the lack of in-depth understanding of the concepts of stigma and discrimination in this setting, and the need for effective stigma reduction interventions that are contextually designed and involve all key stakeholders (service user, service providers, community representatives, service developers and policy makers) (Girma et al., 2022).

This work is a part of the International Study of Discrimination and Stigma Outcomes (INDIGO) project, a long-running global network of initiatives in stigma reduction, which gave rise to the INDIGO Partnership research program, implemented in five countries (Gronholm et al., 2023). This study aims to explore on what the experiences and perceptions of individuals with MHCs regarding stigma are, and how these perceptions looks across different stakeholders, including family members and key stakeholders within mental health services in the Ethiopia context, can be addressed.

## Methods

### Study design and setting

A descriptive qualitative approach was used to obtain an in-depth understanding of the local context based on the viewpoints and perspectives of those who reside and work in the research area. This study was conducted in the south Sodo and Sodo districts of Ethiopia, which are 84 km south of Addis Ababa, the capital of Ethiopia. The district had an estimated total population of 189,970 people as of 2021, as projected by the Ethiopian Central Statistics Agency. The district has 58 health posts, 8 health centers and a primary hospital. Within Ethiopia’s three-tier health system, these facilities offer primary level care. Ethiopian society is largely religious (Mihretu, 2019). The most popular method of treating mental illnesses in Ethiopia is holy water, which is used by Orthodox Christians (Hannig, 2013). This area has been a population and study base for essential health research and intervention a collaborative program between the then department of Community Health (now School of Public Health), Faculty of Medicine, Addis Ababa University for the past 38 years. The findings of this study will be used as input for the preparation of locally tailored anti-stigma programs prevention.

### Participant selection

A purposive sampling technique was used to identify respondents for the in-depth interviews. The predetermined characteristics of the selection participants included the following: key stakeholder groups in the community were people with lived experience (PWLE) of MHCs, family caregivers, health professionals and staff members working in the healthcare setting. Gender and various religious groups were also taken into account. Health facilities in the Sodo district were first contacted by the Addis Ababa University-led INDIGO partnership program to identify volunteer candidates who met the eligibility requirements. These included people who have received a medical diagnosis of an MHC, as well as their caregivers, and those who intended to live in the program region for the duration of the upcoming anti-stigma intervention implementation period (as the same individuals were required for the anti-stigma intervention to be implemented). Health professionals who are active participants in mental health departments from different health facilities located in the Sodo district area were also included. Following this, a team from the Ethiopia site specifically approached the qualified participants to verify their voluntarism, ensure that they are not being coerced into participating in the program, and give them a brief overview of the study plan, project, and other pertinent information. They also informed them of their right to withdraw from the program at any point during data collection. The target MHCs for this study incorporate those major mental health cases in Ethiopia and identified in the Mental Health Gap Action Programme module and: these are depression, psychosis, bipolar disorders and alcohol use disorder.

### Procedures

A total of 16 participants were involved in the study. The number of participants was determined after the data saturation level was achieved during data collection and coding. New data points were found to be replicating patterns that already existed without significantly adding new information. The interviews took place over one or two sessions, depending on participant preference and availability, with each session lasting about 40–50 min using an Amharic-language topic guide. The INDIGO local (Ethiopian) researchers site team conducted semi-structured interviews which adapted and translated into an Amharic version from the predefined frameworks which contains questions about participants’ experiences with stigma and discrimination as well as explanatory models of MHC and local concepts related to interventions against stigma and discrimination. All collected data were audio recorded. All authors participated in the design of the study and development of interview guides and the coding framework.

### Data analysis

The adopted interview guide was translated in to Amharic language. All collected data were coded and transcribed verbatim in Amharic and translated into English by Ethiopia site team (EG, BA, AH). Inter-coder agreement was checked using a sample of transcriptions and inconsistencies were discussed so that the different coders reached to the same level of understanding of each code. Following this, the data analysis for this site specific paper done by EG and BA. The data were analyzed deductively from coding frame that derives from two sources: (1) The explanatory model framework developed by Arthur Kleinman (Kleinman, 1980) and (2) the “What matters most” anthropological concept of understanding stigma (Yang et al., 2014a; 2014b; see Supplementary Table S1 for detail that formed a base for organizing thematic analysis and as a foundation for topic organization). In this step, related concepts from the fundamental themes were grouped together into an organizing theme, which summarize the concepts into a structured format. As a result, the description was completed in accordance with the thematic areas. The credibility of the results was maintained by peer debriefing, and prolonged participation. To ensure reliability peer review is conducted in terms of dependability, the consistency was checked in the findings. A detailed description, of place, context and culture in the research process helped the transferability of the study.

## Results

The 16 participants were mental health service users (PWLEs), caregivers, heads/health administrative leaders, psychiatric nurses, public health officers (see Table 1).

**Table 1.** Demographic characteristic of study participants

No.	Type of participant	Gender	Age	Level of education	Religion
1.	Service user	Male	73	Elementary school	Ethiopian Orthodox
2.	Service user	Male	40	Elementary school	Ethiopian Orthodox
3.	Service user	Female	43	Not educated	Protestant
4.	Service user	Female	23	Secondary education	Ethiopian Orthodox
5.	Service user	Female	39	Secondary education	Ethiopian Orthodox
6.	Caregivers	Male	60	Not educated	Protestant
7.	Caregivers	Male	32	Elementary school	Ethiopian Orthodox
8.	Caregivers	Female	57	Elementary school	Ethiopian Orthodox
9.	Caregivers	Female	52	Secondary education	Ethiopian Orthodox
10.	Heads/health administrators	Male	48	MSc	Ethiopian Orthodox
11.	Heads/health administrators	Male	53	MPH	Ethiopian Orthodox
12.	Service provider	Male	36	Health officer	Ethiopian Orthodox
13.	Service provider	Male	29	Psychiatric nurse	Muslim
14.	Service provider	Male	31	Psychiatric nurse	Ethiopian Orthodox
15.	Service provider	Male	27	BSc nurse	Ethiopian Orthodox
16.	Service provider	Female	24	Health officer	Protestant

The findings are presented under four major themes, (i) explanatory model of stigma and discrimination, (ii) what matters most, (iii) different types of stigma and discrimination and (iv) local concepts toward the interventions on anti-stigma and stigma-related barriers.

### Explanatory models of stigma and discrimination

This describes the perceived and experienced explanatory conditions of stigma and discrimination, encompassing explanations of name or label, manifestations, causes and help seeking.

#### Labels and terms used

A range of derogatory and stigmatizing terms for people affected by severe MHCs (e.g., psychosis) were identified.

By far, the most commonly used term by all groups of study participants was “***Ebde”,*** which refers to a “mad person” or “crazy person”. Most often, this name is given to those people who walk on the street without clothes or display overt manifestations of severe MHCs. The name “***Qews”*** was also widely mentioned by respondents and frequently stated by service users, referring to those who cannot control their actions. The other labels, mostly reported by mental health service users and caregivers, were ***“Nic”*** means that the patient’s brain is injured (the patient’s brain is not functioning properly), ***“Ye AymeroChinqet”*** (mental disturbance) and ***‘Gemash Amanuel”*** (indicating “crazy” or “mentally unstable; the name “Amanuel” refers to a well-known psychiatric hospital which is heavily stigmatized). The other identified local name or term used specifically in Gurage zone, where the data collection took place, was ***“Shiregna”*** (Guragegna language to mean ‘mad person’).

A number of service users mentioned that they did not know these commonly used names given to MHCs and one service user indicated that he did not know the name of the condition affecting him, stating the reason as:

*“Maybe they didn’t want to tell me not to make me worried”. (Male service user, alcohol use disorder, Ethiopian orthodox).*

From the service providers’ side, the majority indicated that the most commonly recorded cases were psychosis, epilepsy and depression. A particular participant stated that:

*For depression the people usually **say “Jezbual”** (became lazy) and for bi-polar patients (manic phase) say **“Neklual”** or **“Tesh Argual”** (meaning that the person could not think normally or clearly)* (Service provider, Male, Muslim, Psychiatric nurse).

#### Manifestations

This section shows people recognize mental health problems and the types of stigmatizing attitudes and behaviors toward PWLEs.

According to the participants, harming others, throwing stones, being seen chained in public, disturbing the neighborhood, trying to flee from home or holy water or other religious places, and walking naked in the street were highly lead to negative assumptions about the person and stigmatization by the community. From the perspective of service users, the most commonly mentioned features were trying to attempt suicide through hanging themselves or drinking poisonous substances, losing consciousness and doing things unknowingly. These were all perceived to contribute to being identified as a ‘mad person’ and stigmatized by society. The majority of participants identified manifestations like excessive talking (even though the person felt normal), lack of sleep, decreased appetite and hallucinations (particularly at night), and tended to make generalizations about people with MHCs, characterizing them as causing disturbances and as violent people.

*The family members give us other information; like, that the patient is disturbing, they were trying to fire the house, they do not sleep well, they talk too much, they tried to escape at night from Holly water or other religious places (Service provider, Male, Ethiopian orthodox, Health officer).*

The level of stigma toward people with MHCs was classified by respondents as falling on a dimension ranging from those who were called “crazy” to those who were “calm” (please see preceding section on “labels and terms” for local language terminology). Included within the commonly mentioned “crazy” designation were those who were perceived to create a disturbance in the surroundings, were violent toward other people, or were not appropriately clothed or ‘spoke suddenly and seemingly without their own will’ or were observed talking to themselves. In general, these people were considered to have been possessed by an ‘evil spirit’.

*“She showed the signs of an evil spirit, speaking out of the blue like other people do when they are possessed by one, so I took her to the holy water (healing approach linked to Orthodox Christian churches).” (Male, Ethiopian orthodox, Caregiver).*

*“…Transportation might not be available and the patients might not be calm and they will create disturbance to bring mentally ill patients from rural to urban area for treatment…” (Male service user, Ethiopian orthodox, Alcohol use disorder).*

The “calm” description included those people with MHC who were emphasized to have acceptable behavior and speech. There was an implication that being in the “calm” group was preferable for individuals to lessen stigma. In this quote, the caregiver appears to be trying to emphasize that their loved one does not fall under the ‘crazy’ category.

*“…My sons do not use violence, or insult others. They are simply too quiet, and people notice that they don’t bother or beat anyone …” (Female caregiver, Ethiopian orthodox).*

#### Causes

This section outlines the possible explanations of the cause of MHCs and the reasons claimed for the use of the stigmatizing terminologies. The most frequently proposed cause of MHCs raised by almost all groups of participants was a supernatural or religious nature. Many did not perceive MHCs as a disease, but rather as the result of bad spirits, witchcraft, and being cursed because of his/her and/or family’s inappropriate actions or sinful acts. Others believed that people were poisoned by harmful traditional drugs given to them by either friends or enemies.

*“Yes, in our society, majority of people have limited knowledge and awareness on mental illness. Therefore, do not consider mental illness as a disease, rather they believe it is related to bad spirit. Many families or caregivers take the patients to religious places nearby most of the time “Tsebel”” (Holy water, since Orthodox religion dominates in the area). (Male service provider, Ethiopian orthodox, Nurse).*

Stigmatization and discrimination against people with MHCs were associated with assumptions about the cause of illness. In this analysis, the other suggested causes were alcohol addiction, use of Khat (*Catha edulis*; a plant chewed commonly in East Africa and some Arab countries that has amphetamine-like properties), or other types of substance use. People were reported to have various reasons for starting to use substances that were also believed to be associated with the development of MHCs. The main ones stated by the participants were loss of family members/close relatives, failure at school and loss of business or being robbed of their property. All these were considered as stressors that could lead either to addiction or directly to MHCs. This explanation was primarily raised by the service providers.

*In cities like Butajira and Welkite (cities where the drug “Khat” is abundant) you will find more people with psychosis on the road trying to gather “Khat” leftovers. It will not be hard to judge that most of the people searching Khat are psychotic patients; even some of them are totally naked. (Male service provider, Psychiatric nurse Muslim, Hospital).*

The other explanation was poverty or living in low socioeconomic status, which was similarly associated with stressors and having limited access to healthcare services. In some cases, caregivers confined those with a severe MHC at home and chained them because they could not afford the treatment costs.

#### Help seeking category

Stigmatized groups tended to seek or expect help from different parts of the community. Service users and caregivers preferred to look for a solution from religious institutions first, for example, “Tsebel” (Holy water in Orthodox religion) or to a Quran house (for Muslims), and considered health facilities to be the last resort most of the time. If the person’s condition did not improve, the caregivers would take them to other more well-known holy water site and stay there for weeks or even months. Being told to stop taking prescribed medicine and only being treated with holy water are some issues with religious settings that prevented people from receiving or accessing treatment.

The Protestant Christian religion was reported to pray for the person and claim that the person was cured, thus discouraging help seeking from the health system. While Muslims engaged in practices like gathering religious leaders, relatives and elders together, chewing “Khat” and praying for the person, then saying that they are healed and do not need to take medicine any more. Another place to seek healing was from traditional healers or sorcerers.

*Instead of bringing the patient on early stage of the illness they prefer to take them to traditional healers or to “Tenquway” (sorcerers) or take them far from their community. Then seeking health care service after it becomes chronic (service provider, male, Ethiopian orthodox, health officer).*

Another frequently noted source of help and support was from friends. Many service users stated that they received advice, support/encouragement and motivation to stay strong from their friends.

*“My friend helped to rent house and start treatment in Butajira hospital. She and her husband advised and helped me financially.” (Female service user, Protestant, Psychosis).*

Additionally, there was support from family members and sometimes from others in their community. Some respondents expressed that the preferred places to seek help was also from healthcare facilities and healthcare workers, (Butajira Hospital, Buei Hospital and Amanuel mental hospitals were frequently mentioned healthcare facilities). The community-based health extension workers were reported to play an important role in creating community awareness, tracing and bringing people with MHCs to health facilities, carrying out follow-up and being friendly with caregivers and encouraging people with MHCs to take their medication.

*“There is good work done by health care centers in collaboration with health extension workers. Those who are staying at home are well taken care of by health extension workers during their house visits and routine follow ups.” (Male, Ethiopian orthodox urban hospital head)*

#### Predominant stereotypes and attitudes

Prevailing community attitudes toward people with MHCs included the following: mental illness could not be cured (with medicine or any other means), mental health illness can be transmitted and the condition is hereditary, and all people with MHCs are assumed to be dangerous and violent. In addition, among service providers, there was a concept of shared/contagious psychosis: that the healthcare worker may get sick from long exposure to people with health conditions:

*“As a fresh health worker, I used to fear that maybe the “shared psychosis” is true and that I should change working in the department of mental health services” (Male service provider, psychiatric nurse, Ethiopian orthodox).*

### What matters the most

The service user and caregiver described expectations in relation to functional role and socially significant matters.

#### Functional role and what matter most

The perceived functional impairment or dysfunction associated with stigmatized identity is described in this section. Respondents highlighted being unable to contribute to important and expected social activities, like ***Idir* (**traditional self-help association that assists grieving families financially, materially and emotionally at the time of a death**)** and ***Mahiber*** (an association or grouping of people with social responsibilities existed in different cultural, religious and socioeconomic contexts) as key indicators of dysfunction that mark out those with MHCs. Inability to complete school, which is more of an issue for younger people with MHC, the inability to find a job and make a living, and the fear of social interaction, were also cited by respondents as problematic. Additionally, being impaired in taking care of oneself, including not knowing how to wash one’s body or dressing oneself, and particularly female participants finding it difficult to take care of household and family responsibilities were salient aspects of functionality according to study participants.

*“I think the community expects me to take care of myself, but I couldn’t take care of my family and control my house and to have job” (Female service user, Ethiopian orthodox, Depression).*

### Type and sources of stigma and discrimination

The different types of stigma experienced by service users and caregivers included (i) self-stigma; (ii) courtesy stigma and (iii) public, institutional and professional stigma.

#### Self-stigma

Participants reported experiencing a feeling of shame and blaming themselves which sometimes led them to refuse to take their medication, isolate themselves from society, stop engaging in various social activities, avoid social interactions and potentially resulted in suicidal ideation and wanting to disappear from their locality and from the community as a result of feeling bad and lonely.

*I caused problem in my family. But I was thinking about attempting suicide and wanted to escape from my home and from the community. (Female service user, Ethiopian orthodox Psychosis).*

#### Courtesy stigma

This reflects the experience stigma and discrimination of caregivers and service providers because of their association or relationship with people with an MHC.

A female caregiver urban house stated,

“*I rented the house using another person’s name and paid monthly using that person. We moved in at night when the owners were out of the city so that they would not notice”. (Female caregiver Ethiopian orthodox).*

#### Public, institutional and professional stigma

The following section presents the stigmatizing behaviors that the targeted group experiences and anticipates. Service users faced isolation and discrimination from the community (not wanting to go to their house and unwilling to be visited by them), locking them behind doors and holding them in chains by their caregivers/family because of their illness (fear they may come to harm or cause trouble) and most of the time to avoid shame. There were also reports of exclusion from public transportation, as it was anticipated that the illness would cause some disturbance.

*I used to be isolated and discriminated by my neighbors and by my husband’s relatives. They do not want me to go to their house and they did not come to visit me either. My neighbors did not allow me to participate in social activities and I felt bad and felt lonely. Even they will not accept my thoughts and words seriously since they think am crazy. (Female service user, Ethiopian orthodox, depression).*

With regard to institutional stigma, for those attending healthcare facilities, an unwelcoming environment for mental health service users was reported, especially in governmental health facilities compared to private institutes and poor law enforcement trend as not treating people with MHCs respectfully and may disregard their complaints. Lack of resources and essential mental health medications, disorganized departments and shortage of health professionals in the psychiatric department were also reported.

“*Yes, there are some challenges especially on the law enforcement, if the patients cause harm the police officers request us to report the patients follow up history in order to confirm if they are mentally ill or not. Nevertheless, most of the time they do not use our report as evidence. Even sometimes the police insult them, they do not accept their complaints”. (Female service provider, protestant, health officer).*

Healthcare professionals were also reported to show stigmatizing behaviors toward service users by refusing to engage in conversation with them, not giving them directions in the health compound, giving other patients who are seeking treatment for physical illnesses priority, mistreating them and failing to give them the attention they need.

*“Once I went hospital to get birth and I was treated differently than other delivering mothers. The nurses were asking how they and their newborn were doing but no one asked me that and I feel sad and cried. I was asking myself why they ignored me.” (Female service user, protestant, psychosis).*

Service users reported, unable to seek medical attention from healthcare providers or withdrew from follow-up appointments and medication, fueling loss of hope, feelings of frustration and even suicide attempts.

Among healthcare professionals specific to mental health, the workload (long working hours) and lesser training opportunities compared to other health disciplines made them susceptible to frustration and discouragement. This was also considered a concern because inadequate training and support could result in incorrect diagnoses and failures to rule out potential conditions, as well as failures to administer the appropriate dosages of medication.

*“Other than that through my five years working experience, there was no trainings initiated by the government. So, it is difficult to update myself and I sometimes question myself if I am giving the patients the appropriate and updated treatment”* (*Service provider, psychiatric nurse, Muslim.*

### Local concepts related to evidence-based interventions against stigma and discrimination

#### Social contact

Most respondents suggested safe spaces and good settings to make social contact are; where the person resides, which could be in their surrounding or in religious places, a village meeting where the local leader facilitates, the government meeting halls like the district administration hall and the city administration halls that can be easily accessible. A particular participant recommended that:

*“If better comfort is needed there is a great private meeting hall in Butajera and it also could be at hospital own meeting hall.”* (*Male service user, Ethiopian orthodox, Alcohol use disorder).*

#### Acceptable contact

Different participants provided their preferences on how and with whom the healthcare worker social contact training should be carried out. According to participants, the majority preferred or would be willing to be trained with the community, implying a chance for service users to explain about how they were treated, what they feel and their need from healthcare providers, so that they would not repeat the mistreatment. In addition, to support implementation, they recommended to assign budget or simple refreshment in order to create awareness in the community.

Others supported the involvement of people with MHCs who had recovered in training courses, because of they can explain better about MHC than non-patients or healthcare providers can, which will help the community to build trust on the healthcare facility, and motivate and boost the person’s confidence.

Some suggested for the training to be held separately, indicating that the level of understanding varies and the need for preparatory work with health workers ahead of social contact interventions.

*“As far as I saw these patients are usually not educated. So I do not think health professionals and mentally ill patients could understand the training equally” (Male service provider BSc nurse, Ethiopian orthodox).* In addition, a particular service provider indicated that training is not needed at all,

“*I do not think training is needed; rather regulations from government are necessary in the area” (Male, service provider, Ethiopian orthodox, health officer).*

**Figure 1.**
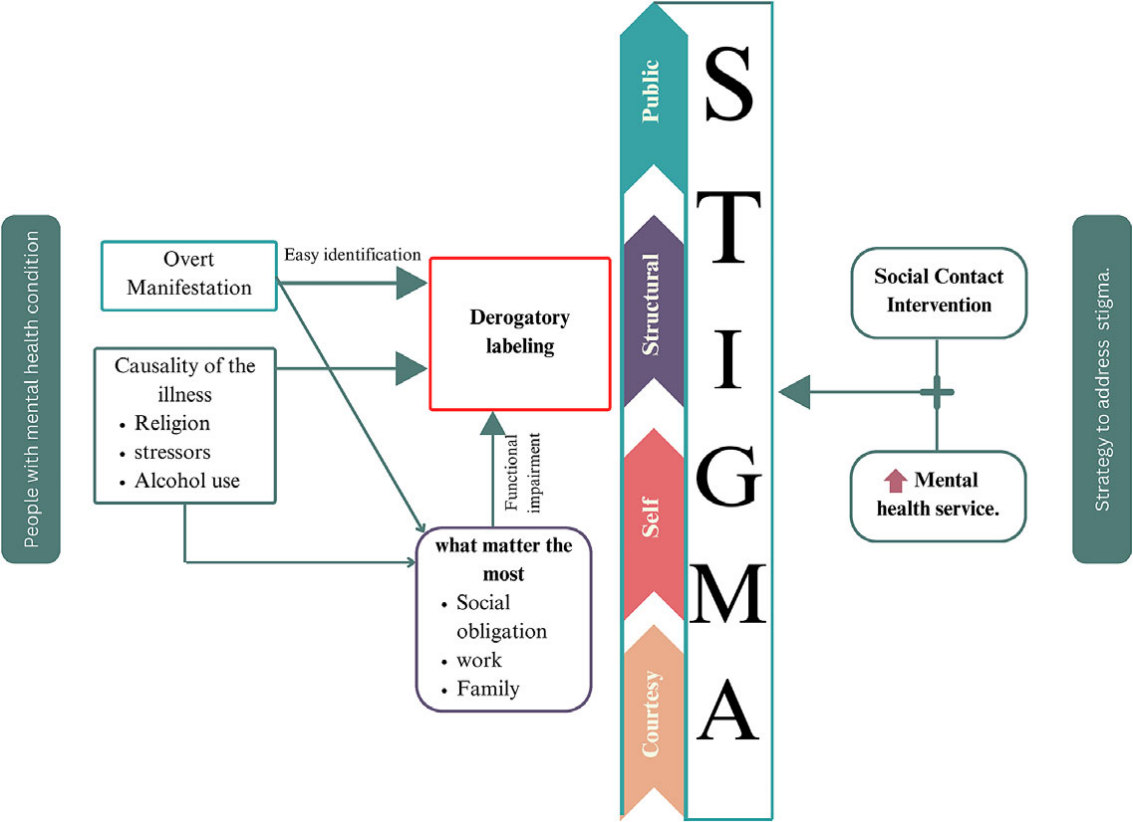
Explanatory models, types of stigma and strategies to combatmental illness stigma and discrimination, Ethiopia, 2024.

The following figure summaries people with MHCs in the Ethiopian setting. Various forms of stigmatization which is linked to attributions about the causality of the illness (mainly related to religious factors, alcohol use and stressors), overt manifestations of MHC (often untreated) leading to easy identification and functional impairments that leads to derogatory labeling adversely affect participation in what is most important to the community (social obligations, family and work). The findings also indicated that social contact is a suitable and relevant intervention strategy to address this different form of stigma when it adds up with increasing access of mental health service (Figure 1).

## Discussion

This study sought to explore key stakeholders’ perceptions and experiences about mental health-related stigmatization in the rural Ethiopian context. The findings indicate that people with MHCs, together with those who care for them, experience stigma in diverse ways as a consequence of the different manifestations of their illnesses, the various labels that have been assigned to them, and beliefs and attitudes of the community that adversely impact their lives in social and individual domains.

In psychiatric practice, mental healthcare professionals use labels based on international diagnostic systems, for example, the Diagnostic and Statistical Manual of Mental Disorders guide, whereby mental “illnesses” have well defined categories and criteria to identify the particular syndrome or illness a person is experiencing (Black and Grant, 2014). However, some people in the community generally refer to people with MHCs with derogatory terms or stigmatizing labels. A study in India stated that the term or adjective that the family uses most frequently *Aalsi* was the laziest, followed by *sustt* and for lethargy and the two terms/adjectives that friends or coworkers most frequently used were *paagal* (mad) and *darpok* (coward) (Grover et al., 2020). Our study in Ethiopia found that, people with MHCs are generalized as violent and disturbance and that various negative labels tended to link to particular types of MHC. Laziness and becoming slothful to depression, and for the severe mental disorder related to “crazy”, becoming insane and losing one’s mind or lack of self-control.

Stigma and social identification are closely related (Klik et al., 2019). Symptoms of mental illness as aggression and nakedness in the public leads to identify people with mental illness as distinctive and make them vulnerable to stigma from the general public (Kimotho, 2018). Greater symptom severity of the mental illness makes people more susceptible to the negative impacts of expected stigma (Fox et al., 2018). This might hold especially true in the context of this study mainly where treatment coverage is low and most people with MHCs have not received care. Stigmatization of people with MHCs was also very likely to result from severe manifestations of MHCs, or behaviors perceived as causing trouble. Poor access to mental healthcare reinforces this stereotype because MHCs are not treated early or adequately and symptoms risk escalation. A previous study conducted in the same rural districts demonstrated that stigma and discrimination reduced when mental healthcare was made locally available integrated within primary healthcare (Tirfessa et al., 2020). This underlines the importance of combining anti-stigma interventions with increased access to mental healthcare.

The perceptions of what causes MHCs are mostly related to cultural ideas about MCHs. a study on perceptions of mental health condition in Ethiopia shows religious/spiritual, disaster and economic deprivation, substance abuse were the most frequent response regarding perceived causes of MHCs as depression and anxiety (Monteiro and Balogun, 2014). This was also reiterated in this study, religious causes (curses, possession by spirits), and addiction to alcohol, Khat and stressors such as loss of family member/close relatives, loss of employment and poor socioeconomic status were mentioned as causes of MHCs.

Religious rituals were the first choice for care for people with MHCs in this study, and in previous studies from Ethiopia (Kasa and Kaba, 2023). Adult users of holy water had a prevalence of common mental disease (Belete et al., 2023). Our finding highlighted that most service users and caregivers prefer to first turn to religious institutions for a solution, to the Quran home or to “Tsebel,” the Holy Water of Orthodox religion, with medical facilities being the last resort. Such evidence was also found, in Jordan, employing ’Rukia,’ praying, and reading the Holy Quran or other religious books could help heal mental illness; with such beliefs related to higher stigma for both people with MHCs and mental health in general (Al-Rawashdeh et al., 2021).

People with severe MHC were more likely to have functional impairment due to the severity of their manifestations, internalized stigma and ongoing illness (Habtamu et al., 2018). In rural parts of Ethiopia, the most highly regarded functional impairment domains, closely linked to what matters most to the community, include the capacity to carry out social obligations, maintain self-care, participate in family life and perform productively at work (Habtamu et al., 2015). In this study, we also identified that people with MHCs were less likely to engage in expected roles and what was considered most important to the community such as to be able to participate in social activities, finding a job, finishing school, taking care of oneself and leading the family, thus making MHCs a potent source of stigma.

Along with the actual MHC, self-stigma is one of the biggest obstacles that people with MHCs must overcome. Lower levels of adherence to treatment are linked to a higher degree of self-stigma (Assefa et al., 2012; Carrara and Ventura, 2018). Our findings also support that self-stigmatization leads people with MHCs to neglect their medication intake and experience social isolation.

Public stigma, or how the general public feels about a group that has been stigmatized, can be understood as consisting of three different components (Corrigan, 2016; Thornicroft et al., 2016). Initially, there may be a problem of knowledge about mental health or mental illness. The second is a negative emotional response to a person with a mental health problem, namely prejudice that significantly impacted negatively by a person who are stigmatized, which also enhances people’s desire for social isolation (Angermeyer and Matschinger, 2003). Discrimination is a third way that prejudice manifests itself behaviorally (Angermeyer and Matschinger, 2003; Corrigan, 2004). Our study participants were noted to be excluded from society; emphasized as “staying at home,” and, in regards to discrimination social exclusion and limitation on using public services like transportation were also evident in this study.

In terms of institutional stigma, there was evidence of an unequal allocation of resources between physical and mental health at different levels of the healthcare system, particularly in regions where there is a dearth of mental health service providers. This has been reported in other settings, including a relative lack of resources, access to vital medications and personnel concerns differentially affecting mental healthcare (Pugh et al., 2015; Thornicroft et al., 2022). This study also found that both service users and providers were impacted by the government’s lack of attention to the mental health sector, which was evident in the lack of capacity building of healthcare professionals, in not hiring mental health professionals, inadequate provision of medications and in the lack of comprehensive care (including psychosocial care). People with MHCs were rendered unable to seek medical attention from healthcare providers or withdrew from follow-up appointments and medication, which contributed to losing hope, feeling frustrated and even attempting suicide.

## Limitation

While this study evaluated some important stakeholders in mental health, it did not consider the stigma within the larger population including religious leaders and traditional healers and was restricted to a specific subset of healthcare practitioners.

## Conclusions

To conclude, our findings have implications for both measurements of stigma and the development of interventions. Measures need to tap into contextually important aspects of stigma. The focus of respondents on the behavioral consequences of stigma, particularly exclusion, might support the use of measures that focus on discrimination rather than attitudes. In terms of intervention, social contact was seen as relevant and an acceptable approach, likely to be most impactful if the individual was from the local community. Alongside social contact, it is essential to create an enabling environment, with training of service providers in working collaboratively with service users, raising community awareness and (crucially) expanding access to mental healthcare in the local community.

## Supporting information

Girma et al. supplementary material Girma et al. supplementary material

## Data Availability

The data that support the findings of this study are available from the corresponding author, (EG), upon reasonable request.

## Abbreviations

AUD: Alcohol use disorder
PWLEs: People with lived experiences
MCHs: Mental health conditions

## Supplementary material

The supplementary material for this article can be found at http://doi.org/10.1017/gmh.2024.55.
