# Supplementary material for: Understanding mental health stigma and discrimination in Ethiopia: A qualitative study
Source: Glob Ment Health (Camb). 2024 Apr 30;11:e58. doi: 10.1017/gmh.2024.55 (PMC11362999; doi:10.1017/gmh.2024.55)
Supplement: Girma et al. supplementary material [file S2054425124000554sup001.docx]

**Supplementary file**

**Table 2: Code book from INDIGO partnership program formative work.**

| **Sr. no.** | **Codes** | **Description of the codes** |
| --- | --- | --- |
| 2 | Categorization of stigmatized group | Describe the group being stigmatized from the perspective of group members, stigmatizing group, and general public) |
| 3 | Name | Any term used in the society/culture to label mental health problem/shared term/language |
| 4 | Manifestations | The meaning or description of terms used to label mental problems |
| 5 | Cause | Reasons for using the terms |
| 6 | help seeking | The process of help seeking e.g. when did the problem detected, how it was known, where they went first, challenges in help seeking… |
| 7 | Myths | These are any beliefs from the different groups (stigmatized, stigmatizer, general public) that are false beliefs related to the stigmatized group (e.g., contagion for non-contagious conditions, not-recoverable, can’t touch people, etc.)  The section also includes negative attitudes towards the stigmatized group such as desire for social distance and emotional reactions of fear, anger or pity |
| 8 | Other characters | Describe any other characteristics typically associated with the stigmatized group—this reflects the concept of ‘intersectionality’ in stigma work, e.g., race and mental health, poverty and mental health, sexual orientation and mental health, etc.; here also describe based on the stigmatized group’s perspective, stigmatizing group, and general public |
| 9 | Functioning role | Describe the perceived functioning or functional impairment associated with the stigmatized identity; e.g., work productivity, family role, educational productivity, self-care, etc. |
| 10 | Matters most | Responses in relation to the expectation of what is important for a man/ woman. E.g. job, money, marriage… |
| 11 | Expectations | Expectations of stigmatized group towards the treatments or other intervention approaches |
| 12 | stigmatization location | The setting where the stigma and discrimination did occur. E.g. at home, health facility, neighborhood… |
| 13 | stigmatizing behavior | Types of stigmatizing behavior. E.g. the difference in receiving the services in the facility, the reaction of people when they seek help… |
| 14 | structural stigma | Structural facilitators and mitigators of stigma E.g. reasons for mistreatment, activities to Minimize ill treatment... |
| 15 | safe contact | Safe spaces of social contact. E.g. the good setting to make social contact like the health facility, community, schools or institutions |
| 16 | acceptable contact | Types of acceptable social contact E.g. their role during the training, their preference on the training approach |

**Table 3: Consolidated criteria for reporting qualitative studies (COREQ): 32-item checklist**

| **No.  Item** | **Criteria description** | **Study Information** |
| --- | --- | --- |
| **Domain 1: Research team and reﬂexivity** |  |  |
| *Personal Characteristics* |  |  |
| 1. Interviewer/ facilitator | Which author/s conducted the interview or focus group? | Two qualitative researchers with master’s level education |
| 2. Credentials | What were the researcher’s credentials? E.g. PhD, MD | Ethiopia: PhD, MPH |
| 3. Occupation | What was their occupation at the time of the study? | Ethiopia: Associate Professor of Health Promotion and Health Communication, School of Public Health, Addis Ababa University, Ethiopia  Project Manager, College of Health Sciences, AAU  President of the Ethiopian Health Education and Promotion Professionals Association (EHEPA)  USA: Research associate & Dr PH student at George Washington University; Program manager at National Institutes of Health & Dr PH student at George Washington University |
| 4. Gender | Was the researcher male or female? | Ethiopia: Three female, Two male  Nepal: Male |
| 5. Experience and training | What experience or training did the researcher have? | Ethiopia: 10+ years of experience in qualitative methods  USA: 8 years’ research in neuropsychology and global mental health, with two years’ experience conducting qualitative research and 6 months of qualitative training courses; Long-term experience in quantitative research in environmental and occupational health, but limited research experience in qualitative research. |
| *Relationship with participants* |  |  |
| 6. Relationship established | Was a relationship established prior to study commencement? | No prior relationship |
| 7. Participant knowledge of the interviewer | What did the participants know about the researcher? e.g., personal goals, reasons for doing the research | A minority of participants knew of the interviewers’ work affiliations and all were briefed about the study purpose, none had information on personal goals |
| 8. Interviewer characteristics | What characteristics were reported about the interviewer/facilitator? e.g., Bias, assumptions, reasons and interests in the research topic | Ethiopia: Interest in mental health and stigma topics |
| **Domain 2: study design** |  |  |
| *Theoretical framework* |  |  |
| 9. Methodological orientation and Theory | What methodological orientation was stated to underpin the study? e.g. grounded theory, discourse analysis, ethnography, phenomenology, content analysis | A descriptive qualitative approach was used to produce an in-depth understanding of the local context that is based on the viewpoints and perceptions of those who reside and work in the research area.  The study used a combination of deductive and inductive methodology in a thematic framework analysis approach for data collection and analysis. |
| *Participant selection* |  |  |
| 10. Sampling | How were participants selected? e.g. purposive, convenience, consecutive, snowball | A purposive sampling technique in order to selection information-rich cases related to the interest of the study |
| 11. Method of approach | How were participants approached? e.g. face-to-face, telephone, mail, email | Face to face interviews take place over one or two sessions. |
| 12. Sample size | How many participants were in the study? | See Table 1 in the manuscript |
| 13. Non-participation | How many people refused to participate or dropped out? Reasons? | None |
| *Setting* |  |  |
| 14. Setting of data collection | Where was the data collected? e.g., home, clinic, workplace | All service provider at their work place and some service users interviewed at health facility and some at home |
| 15. Presence of non-participants | Was anyone else present besides the participants and researchers? | No |
| 16. Description of sample | What are the important characteristics of the sample? e.g., demographic data, date | Table 1 contains demographic information. |
| *Data collection* |  |  |
| 17. Interview guide | Were questions, prompts, guides provided by the authors? Was it pilot tested? | Yes |
| 18. Repeat interviews | Were repeat interviews carried out? If yes, how many? | No |
| 19. Audio/visual recording | Did the research use audio or visual recording to collect the data? | Audio recording |
| 20. Field notes | Were ﬁeld notes made during and/or after the interview or focus group? | Interviews were conducted and immediately translated into English by the interviewers; no field notes or debriefing notes were made. |
| 21. Duration | What was the duration of the interviews or focus group? | With an average 40 to 50 minutes for each session. |
| 22. Data saturation | Was data saturation discussed? | Yes |
| 23. Transcripts returned | Were transcripts returned to participants for comment and/or correction? | No |
| **Domain 3: analysis and ﬁndings** |  |  |
| *Data analysis* |  |  |
| 24. Number of data coders | How many data coders coded the data? | One person was responsible for coding and compiling all of the information obtained from the site. |
| 25. Description of the coding tree | Did authors provide a description of the coding tree? | Yes. |
| 26. Derivation of themes | Were themes identiﬁed in advance or derived from the data? | Inductive coding was used to find additional codes after initially using pre-developed thematic coding. |
| 27. Software | What software, if applicable, was used to manage the data? | NVIVO |
| 28. Participant checking | Did participants provide feedback on the ﬁndings? | The findings were not reviewed by the participants |
| *Reporting* |  |  |
| 29. Quotations presented | Were participant quotations presented to illustrate the themes/ﬁndings? Was each quotation identiﬁed? e.g. participant number | Major themes and findings were illustrated with quotations. For each of these statements, a basic demographic description (participant type, country) was provided. |
| 30. Data and ﬁndings consistent | Was there consistency between the data presented and the ﬁndings? | Yes |
| 31. Clarity of major themes | Were major themes clearly presented in the ﬁndings? | Yes (major headings in the results section) |
| 32. Clarity of minor themes | Is there a description of diverse cases or discussion of minor themes? | Yes (sub-headings in the results section) |

Source: Tong A, Sainsbury P, Craig J. Consolidated criteria for reporting qualitative research (COREQ): a 32-item checklist for interviews and focus groups. *International Journal for Quality in Health Care*. 2007. Volume 19, Number 6: pp. 349 – 357
