# Supplementary material for: Understanding mental health stigma and discrimination in Ethiopia: A qualitative study
Source: Glob Ment Health (Camb). 2024 Apr 30;11:e58. doi: 10.1017/gmh.2024.55 (PMC11362999; doi:10.1017/gmh.2024.55)
Supplement: Peer review history

# Editor report: Recommendation: Understanding mental health stigma and discrimination in Ethiopia: A qualitative study — R0/PR2

**DOI:** 10.1017/gmh.2024.55.pr2

**Affiliations:** Division of Behavioral Health Services and Policies, Columbia University Vagelos College of Physicians and Surgeons, United States

No accompanying comment.

# Editor report: Decision: Understanding mental health stigma and discrimination in Ethiopia: A qualitative study — R0/PR3

**DOI:** 10.1017/gmh.2024.55.pr3

**Affiliations:** London School of Hygiene & Tropical Medicine, United Kingdom of Great Britain and Northern Ireland

No accompanying comment.

# Editor report: Recommendation: Understanding mental health stigma and discrimination in Ethiopia: A qualitative study — R1/PR5

**DOI:** 10.1017/gmh.2024.55.pr5

**Affiliations:** Division of Behavioral Health Services and Policies, Columbia University Vagelos College of Physicians and Surgeons, United States

No accompanying comment.

# Editor report: Decision: Understanding mental health stigma and discrimination in Ethiopia: A qualitative study — R1/PR6

**DOI:** 10.1017/gmh.2024.55.pr6

**Affiliations:** London School of Hygiene & Tropical Medicine, United Kingdom of Great Britain and Northern Ireland

No accompanying comment.

# Editor report: Recommendation: Understanding mental health stigma and discrimination in Ethiopia: A qualitative study — R2/PR8

**DOI:** 10.1017/gmh.2024.55.pr8

**Affiliations:** Division of Behavioral Health Services and Policies, Columbia University Vagelos College of Physicians and Surgeons, United States

No accompanying comment.

# Editor report: Decision: Understanding mental health stigma and discrimination in Ethiopia: A qualitative study — R2/PR9

**DOI:** 10.1017/gmh.2024.55.pr9

**Affiliations:** London School of Hygiene & Tropical Medicine, United Kingdom of Great Britain and Northern Ireland

No accompanying comment.
